# Merging concepts - coupling an agent-based model of hematopoietic stem cells with an ODE model of granulopoiesis

**DOI:** 10.1186/1752-0509-7-117

**Published:** 2013-11-01

**Authors:** Axel Krinner, Ingo Roeder, Markus Loeffler, Markus Scholz

**Affiliations:** 1 Institute for Medical Informatics and Biometry, TU Dresden, Blasewitzer str. 86, D-01307 Dresden, Germany; 2 Institute for Medical Informatics, Statistics and Epidemiology, University of Leipzig, Haertelstr. 16-18, D-04107 Leipzig, Germany; 3 LIFE Research Center for Civilization Diseases, University of Leipzig, Philipp-Rosenthal-Straße 27, D-04103 Leipzig, Germany

## Abstract

**Background:**

Hematopoiesis is a complex process involving different cell types and feedback mechanisms mediated by cytokines. This complexity stimulated various models with different scopes and applications. A combination of complementary models promises to provide their mutual confirmation and to explain a broader range of scenarios. Here we propose a combination of an ordinary differential equation (ODE) model of human granulopoiesis and an agent-based model (ABM) of hematopoietic stem cell (HSC) organization. The first describes the dynamics of bone marrow cell stages and circulating cells under various perturbations such as G-CSF treatment or chemotherapy. In contrast to the ODE model describing cell numbers, our ABM focuses on the organization of individual cells in the stem population.

**Results:**

We combined the two models by replacing the HSC compartment of the ODE model by a difference equation formulation of the ABM. In this hybrid model, regulatory mechanisms and parameters of the original models were kept unchanged except for a few specific improvements: (i) Effect of chemotherapy was restricted to proliferating HSC and (ii) HSC regulation in the ODE model was replaced by the intrinsic regulation of the ABM. Model simulations of bleeding, chronic irradiation and stem cell transplantation revealed that the dynamics of hybrid and ODE model differ markedly in scenarios with stem cell damage. Despite these differences in response to stem cell damage, both models explain clinical data of leukocyte dynamics under four chemotherapy regimens.

**Conclusions:**

ABM and ODE model proved to be compatible and were combined without altering the structure of both models. The new hybrid model introduces model improvements by considering the proliferative state of stem cells and enabling a cell cycle-dependent effect of chemotherapy. We demonstrated that it is able to explain and predict granulopoietic dynamics for a large variety of scenarios such as irradiation, bone marrow transplantation, chemotherapy and growth factor applications. Therefore, it promises to serve as a valuable tool for studies in a broader range of clinical applications, in particular where stem cell activation and proliferation are involved.

## Background

Hematopoietic stem cells (HSC) have been in the focus of research since the beginning of last century [1]. Easy accessibility and handling, in combination with elegant experimental techniques like clonal assays [2,3] made the hematopoietic system the best studied mammalian stem cell system. As a consequence, the first models were designed in the 1960s [4,5]. The process of hematological homeostasis is characterized by a relative stability of the (small) stem cell pool and a massive amplification along the differentiation process, leading to a daily production of about 10^11^-10^12^ mature blood cells [6]. This observation led to the so called pedigree concept, which postulates that stem cells originate only from stem cells, i.e. either maintain the stem cell state or lose it irreversibly [7].

This concept represents a core assumption of most mathematical models for hematopoiesis that have been formulated complementary to experiments. Some do not explicitly model stem cells but include them as a source of cellular influx into the modeled differentiation stages of hematopoiesis [8-10]. Models that explicitly model the hematopoietic stem cell population mostly focus on the cell number of one [11,12], or more populations (such as a resting and proliferating cells [13]. Considering cell numbers these models implicitly ignore inter-cellular homogeneity. A few models do consider structured cell populations and introduce an additional cellular feature [14]. However, all these models share the concept of unidirectional cell flux towards differentiated states.

Following this concept, we also developed ordinary differential equations (ODE) based lineage models of human granulopoiesis, erythropoiesis and thrombopoiesis [15-19]. All these models are supplied by the same stem cell model. They describe the dynamic regulation of HSC, proliferating and maturing progenitors, mature blood cells and cytokines of the hematopoietic system and aim at predicting the complex dynamics of hematopoiesis during combined chemotherapy and growth factor applications. A number of feedback loops control differentiation and amplification, e.g. via the probability of stem cell self-renewal, amplification rates and maturation times of committed cells. Models of pharmacokinetics and -dynamics of growth factor and chemotherapy applications were introduced recently, allowing precise predictions of clinical data in numerous scenarios [19].

The strictly hierarchical pedigree concept was challenged as experimental evidence for stem cell flexibility was found at the end of the last century. Cells from neural [20], skeletal [21,22] and vascular tissue [23] were shown to be capable of engraftment in irradiated hosts and to contribute subsequently to the production of mature blood cells. Most likely this flexibility is induced and controlled by the stem cell environment [24,25]. Our formerly developed agent-based model (ABM) of hematopoietic stem cell organization incorporates such a context dependent stem cell regulation by considering two stem cell growth environments (GE) [26]. In one of these two GE, which can be interpreted as a *stem cell niche*[24], the stem cells are quiescent and regenerate their ability to remain in the niche, while in the other GE they proliferate and lose this niche affinity. The broad range of successful applications of this modeling concept includes modeling of clonal competition [27,28], age-dependent repopulation potential after irradiation [29] and treatment of chronic myeloid leukemia with Imatinib and interferon-α [30-32].

Certainly, the aim of systems biology and modeling is generation of consistent explanations for as many scenarios as possible. Therefore, the following natural question arises from the diversity of individual models and the different scenarios they explain: Can we assume their compatibility and even expect synergistic advantages from their combination? Desirably, the combined models should cover (many) complementary scenarios for maximal synergistic effects, but apply for the same scenarios for compatibility. Due to this premise, we pursue this question with an attempt of second-level modeling: a combination of our ODE model of human granulopoiesis that follows the hierarchical paradigm and our agent-based stem cell model. Both models were developed on the basis of different biological evidence, experiments and data and are well established. Thus our effort was to combine them without changing their assumptions, equations, parameters and application scenarios as far as possible.

If the models prove to be compatible, we can expect the mentioned synergistic effect for a set of reasons. The ODE model turned out to be highly sensitive to changes in its stem cell compartment (SCC) [17]. Exchanging its homogeneous stem cell population for a structured stem cell model enables modeling of phenomena caused by heterogeneity within the SCC, such as lineage priming of stem cells, clonal competition like leukemia development and treatment or cell-cycle specific susceptibility to chemotherapy or radiation. Although ODE model simulations of granulocyte dynamics under chemotherapy agree well with clinical data, predicted reduction of stem cell numbers appears to be over-estimated [33]. On the other hand, the ABM does neither consider effects of growth factors nor their pharmacokinetics. As a result, the ABM is not able to correctly account for short-term effects in the reconstitution of blood cell number after chemotherapy which is often supported by growth-factor applications. Besides, ABM simulations for realistic cell numbers from stem cells to differentiated cells are computationally expensive.

Therefore, we here propose an integration of these model concepts guided by the motivation that systems-biologic modeling is an iterative process. We substitute the ODE stem cell model by a difference equation-based formulation of the ABM describing continuous cell numbers [34]. By this approach, we expect to construct a more comprehensive model with a broader range of possible applications, e.g. in the context of planning of clinical trials.

The adaptations necessary to combine the models will be discussed in detail. We compare the new hybrid model with the former ODE model in a number of qualitative tests, e.g. with respect to chronic irradiation or bleeding. Finally, we used the hybrid model to simulate four different chemotherapy regimens comprising both, dose and time intensifications of therapy and growth-factor applications as well.

## Methods

In the following, we briefly explain our ODE model for human granulopoiesis, our ABM stem cell model and its difference formulation. Building on this background we then establish our hybrid model.

### ODE model for human granulopoiesis

**Figure 1:**
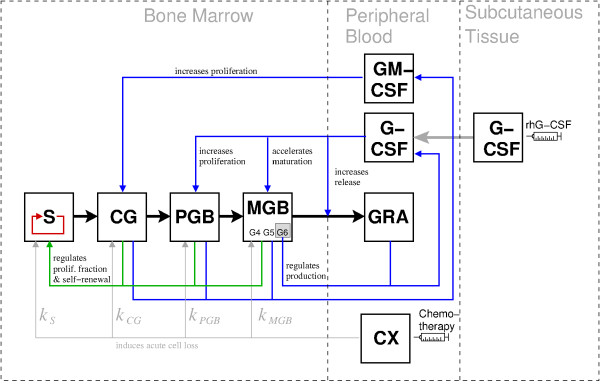
**Schematic representation of the ODE model.** Compartments representing cell types and growth factors are depicted as boxes (s. Table 1). Compartment MGB is substructured into G4-6 to model maturation with Γ-distributed transit times. Cell fluxes between compartments are shown as black arrows, the effect of chemotherapy as gray arrows with associated kill rate *k*_X_ indicated by a label and feedbacks as colored arrows: intrinsic stem cell feedback (red), feedback from bone-marrow cells to stem cells (green) and feedback between later stages of granulopoiesis mediated by explicitly modeled growth-factors (blue). The colored arrows indicate the input for the feedback functions (s. Appendix A2.1) that dynamically control compartment parameters mentioned by the labels.

We here summarize the central features of the ODE model of human granulopoiesis. The complete set of equations and parameters is given in the Appendix (A1.1 and A1.2). All model assumptions are comprehensively discussed in Scholz et al. [17]. The model describes the dynamics of concatenated cell compartments by a set of ODEs (Figure 1). Cell compartments represent cell numbers of morphologically distinguishable cell stages of granulopoiesis, namely HSC, colony-forming units, maturing blasts in the bone marrow and mature granulocytes in peripheral blood (see Table 1) [17]. The two growth factors granulocyte-macrophage colony stimulating factor (GM-CSF) and granulocyte colony stimulating factor (G-CSF) are also explicitly modeled and regulate amplification and transit times in bone marrow compartments.

**Table 1:** List of all compartments in the ODE model and their biological equivalents

**Compartment**	**Meaning**	**Biological equivalents**
S	hematopoietic stem cells	hematopoietic stem cells
CG	granulopoietic progenitor cells	CFU-GM (colony forming units of granulocytes and macrophages)
PGB	proliferating granulopoietic precursor cells	myeloblasts, promyelocytes, precursor myelocytes
MGB	maturing granulopoietic precursor cells	metamyelocytes (G4), banded (G5) and segmented (G6) granulocytes
GRA	granulocytes	granulocytes in circulation
G	CG + PGB + MGB	total granulopoietic cells in bone marrow
G-CSF	G-CSF (granulocyte colony-stimulating factor)	cytokine of granulopoiesis
GM-CSF	GM-CSF (granulocyte, macrophage colony-stimulating factor)	cytokine of granulopoiesis
CX	Chemotherapy	(damaging effect of) chemotherapy

In all individual cell compartments, except for stem cells, a general balance equation reflects the dynamics of growth, differentiation and cell death due to chemotherapy: change in compartment size = influx × amplification - efflux - cell loss.

Influx is given by the efflux of the upstream compartment, while amplification and efflux of compartment *X* are computed from the amplification-related parameters proliferative fraction *a*_*X*_ and amplification *A*_*X*_ and the residence-related parameters transit times *T*_*X*_ (or in compartment S probability of self-renewal *p*). The stem cell compartment represents the root of the hematopoietic tree without influx. In this case, the first term of the balance equation is simply replaced by proliferation/amplification.

Amplification and differentiation is controlled by the four feedback loops sketched in Figure 1. Amplification and self-renewal in the stem cell compartment are regulated by stem cell number *C*_*S*_ (Figure 1, red) and the number of granulopoietic bone marrow cells *C*_*G*_ (Figure 1, green). A decrease/increase in both cell numbers *C*_*S*_ and *C*_*G*_ results in an increase/decrease, respectively, in proliferation, while the effects of *C*_*S*_ and *C*_*G*_ on self-renewal are contrary: low stem cell numbers *C*_*S*_ result in high self-renewal with higher priority than low bone marrow cell number *C*_*G*_ causing increased differentiation. These two feedback functions are adopted from Wichmann and Loeffler (s. Appendix A1.1) [15].

The remaining two feedback loops (blue) control maturation and amplification in the whole bone marrow except for the SCC. They are mediated by the growth factors G-CSF and GM-CSF (s. Appendix A1.1) [35-37]. G-CSF acts in different modes: It increases proliferation, maturation and the release of bone marrow cells to the blood. GM-CSF regulates amplification in CG. Endogenous production of both growth factors depends on cell numbers in the bone marrow and the blood. Subcutaneous and intravenous G-CSF applications were included by a simple pharmacokinetic model. We model a delayed influx from the subcutaneous compartment by a two-compartment system. Degradation in the central compartment is assumed to happen unspecifically and specifically with the specific degradation being proportional to the number of circulating granulocytes. We assumed that applied G-CSF acts in the same way as endogenous G-CSF, i.e. novel G-CSF pharmaceuticals such as pegylated G-CSF with different pharmacodynamics were not considered here.

According to mouse data, chemotherapy was modeled as an acute transient depletion of bone marrow cell stages following a first order kinetics depending on the drugs applied and the cell compartments affected. (s. Appendix A1.1) [33].

All cell compartments are normalized with respect to the setting *C*_*S*_ = 1 in steady-state. Hence, all subsequent compartments are given in units of equilibrium stem cell number. Furthermore, we usually present relative compartment sizes *Cx*^rel^ = *Cx* / *Cx*^nor^. The steady-state values *Cx*^nor^ for all compartments *X* are given in the Appendix (A1.3).

### The agent-based stem cell model

**Figure 2:**
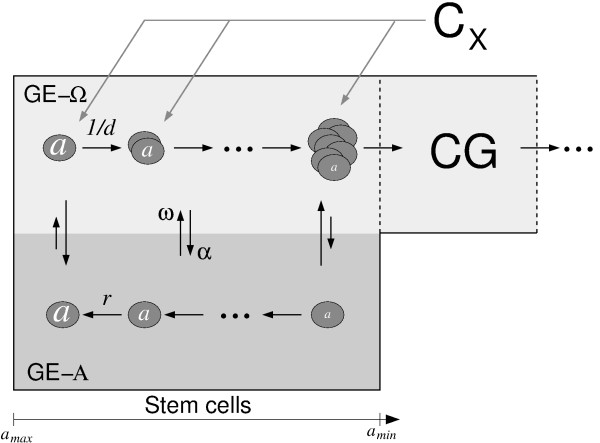
**Scheme of the agent-based stem cell model and its coupling in the hybrid model.** Each cell is characterized by its affiliation to one GE, niche affinity *a* < *a*_max_ and cell cycle position *c*. The two GEs represent functionally different environments: in GE Α affinity *a* increases with time and is limited by *a*_max_. In GE Ω it decreases and cells proliferate. When *a* drops below the threshold *a* = *a*_min_, the cells lose the ability to switch to Α and leave the stem cell compartment. In the hybrid model they enter the progenitor compartment CG. The effect of chemotherapy CX was modeled as cell loss in GE Ω without affecting GE A.

In contrast to the representation of the stem cell population in the ODE model, our agent-based stem cell model represents each cell individually. A general scheme of the model is given in Figure 2. Each cell is characterized by the growth environment GE that accommodates the cell and a niche affinity *a*∈[*a*_min_, *a*_max_ ] [26]. In each simulation step the cells can either remain in or switch between the two GEs. GE Α and Ω represent functionally distinct signaling contexts and may be interpreted as a niche and a non-niche environment, respectively. In the niche environment Α, cells are quiescent and regenerate niche affinity *a*, while in the non-niche GE Ω they proliferate and lose niche affinity. If a cell acquires an affinity lower than *a*_*min*_, it loses its ability to switch back to Α, to regenerate affinity *a* and to self-renew. Then it leaves the stem cell compartment and enters further differentiation and amplification stages. When a cell switches GEs, its affinity *a* remains constant. The evolution of each cell’s affinity *a* at time *t* is given by:

(1) $$a_{t+1}=\left\{\begin{array}{c} \min\left(a_{t}\cdot r,a_{\max}\right)\;\\ a_{t}/d\\ a_{t} \end{array}\right)\begin{array}{l} \mathrm{in}\;A\\ \mathrm{in}\;\Omega \\ \mathrm{for}\;\mathrm{transistions}\;\left(A\to \Omega \right)\;\mathrm{and}\;(\Omega \to A) \end{array}$$

Here *r* > 1 and *d* > 1 are the regeneration and differentiation coefficients, respectively. The cells at *a*_max_ in GE Α conserve their state unless they switch GEs.

The transitions between the GEs happen randomly with transition intensities (probability per time step) that depend on cell numbers in the target GEs and the individual cell’s affinity. These dependences introduce an intrinsic regulation of stem cell proliferation and differentiation. For a cell of affinity *a* and a HSC population of *N*_Α_ cells in GE Α and *N*_Ω_ cells in GE Ω, they are given by:

(2) $$\begin{array}{l} \alpha \left(a,N_{A}\right)=\frac{a}{a_{\max}}f_{\alpha }\left(N_{A}\right)\;\text{and}\\ \omega \left(a,N_{\Omega }\right)=\frac{a_{\min}}{a}f_{\omega }\left(N_{\Omega }\right) \end{array}$$

where α and ω are the intensities of transitions Ω → Α and Α → Ω, respectively. The functions *f*_α_ and *f*_ω_ model a limited capacity of both GEs approaching zero for increasing cell numbers in the target environment. Their definition is given in the Appendix (A2.1).

Because at *a*_max_ the cells are quiescent and the probability of their transition to Ω, and with it activation, is minimal, these cells can be interpreted as dormant stem cells [38].

Proliferating cells in Ω are additionally characterized by their cell cycle position *c*. In each simulation step it is simply increased by the time step: *c*_*t*+1_ = *c*_*t*_ + Δ*t*. The established parameter set for human HSC assumes 48 h for a complete cell cycle, which is divided into two sub-phases: an intermediate G_1_-phase of 32 h (0 h ≤ *c* < 32 h) and a mitotic phase of 16 h (32 h ≤ *c* ≤ 48 h), subsuming S/G_2_/M-phases [32]. During mitotic phase, no transitions to GE Α are allowed. At the end of cell cycle, a cell divides into two daughter cells. They inherit the affinity of their mother cell and start cell cycle in G_1_-Phase (*c* = 0 h). If a cell switches to GE Α, it always becomes quiescent. If a cell switches to GE Ω, it enters the mitotic phase at *c* = 32.

A summary of all parameters of the stem cell model is given in the Appendix (A2.2).

### Difference equation formulation

When considering realistic cell numbers, simulations of individual cells are computationally expensive. To replace the ODE SCC we thus decided to use a difference formulation of the ABM introduced in the last section which was established by Kim et al. [34]. This difference formulation is based on exactly the same equations and parameters as the ABM. It reduces computational effort by defining a discrete set of affinities and describing numbers of cells of those affinities instead of individual cells of arbitrary affinities [34]. Considering Eq. (1) this discrete set is constructed as follows:

1. If a cell in GE Ω starts with maximal affinity *a*_max_ = *a*_0_ = 1 and does not change GE, it will adopt the affinities *a*_*k*_ = *d* ^−*k*^ ; *k*∈ℕ_0_ and eventually leave GE Ω at *a*_min_. Hence, defining $k_{d}^{\max}=\max\;\left({\left\{k|{d^{-}}^{k}>a_{\min}\right\}}_{\mathbb{N}}\right)$, its niche affinities in GE Ω are fully described by the set $k\in \left\{0,1,\ldots ,k_{d}^{\max}\right\}$ with the associated affinities *a*_*k*_ *= d* ^−*k*^ . Analogously, all niche affinities in A can be mapped onto $\left\{0,1,\ldots ,k_{r}^{\max}\right\}$ with $k_{r}^{\max}=\max\left({\left\{k|{r^{-}}^{k}>a_{\min}\right\}}_{\mathbb{N}}\right)$ and the affinities *a*_*k*_ *= r*^*−k*^.

2. If a cell switches GEs, its affinity is conserved (Eq.1) and to set up the difference equations formulation two matching sequences of affinities are required in the two GEs. This is not the case in general, if *d* ≠ *r*. But if ln *r* / ln *d* is a rational number with ln *r* / ln *d* = *v*/*w*, the set of all possible affinities is given by $\left\{{e^{-}}^{\mathrm{k\rho}},k\in {\mathbb{N}}_{0}^{+}\right\}$ with *ρ* = ln *r* / *v* = ln *d* / *w*. The established parameters of the human system are *r* = 1.1 and *d* = 1.05 with ln *r* / ln *d* ≈ 2. Setting *ρ* = 0.0488 results in the slightly modified coefficients *r* = e^2*ρ*^ = 1.10252 and *d* = e^*ρ*^ = 1.05001. Together with *a*_min_ = 0.002 from the human parameter set, this results in *k*_max_ = 127.

Hence, the difference formulation describes the number of cells Α_*k*_ and Ω_*k*_ in compartments that are associated with the affinities

(3) $$a_{k}=e^{-\mathrm{kp}}\;\mathrm{with}\;k\in \left\{0,1,\ldots ,127\right\}$$

While the quiescent cells in GE Α are fully characterized by their affinity *a*, in GE Ω the cellular state is given by the combination of affinity *a* and cell cycle position *c*. The number Ω_*k*_ of cells of affinity *a*_*k*_ in Ω is subdivided into the number of cells Ω_*k,c*_ of cell cycle position *c.* With *c* running from 0h to 48 h and a simulation time step of Δ*t* = 1 h this leads to 49 steps, a cell cycle duration of τ = 49 h and a 128 × 49 matrix Ω_*k,c*_ of cellular states. Thus, the cell numbers *N*_Α_ and *N*_Ω_ that determine the transition characteristics are given by:

(4) $$N_{A}\left(t\right)=\sum_{k=0}^{127}A_{k}\left(t\right)$$

(5) $$N_{\Omega }\left(t\right)=\sum_{k=0}^{127}\sum_{c=0}^{48}\Omega_{k,c}\left(t\right),$$

where *A*_*k*_ is the number of cells of affinity *a*_*k*_ in GE Α and Ω_*k,c*_ is the number of cells of affinity *a*_*k*_ and cell cycle position *c* in GE Ω. For each value of *a*_*k*_, the transition probabilities α and ω are given by Eq. (2). For computation time, we approximated the binomially distributed number of cells that switch GEs with their expectation values ω(*a*_*k*_,*N*_Ω_(t)) · *A*_*k*_(t) and α(*a*_*k*_,*N*_Α_(t)) · Ω_*k,c*_(t) for *c* = 0…31. These settings finally result in the following set of difference equations:

(6) $${Α}_{k}\left(t+1\right)=\left\{\begin{array}{ll} {Α}_{0}\left(t\right)\left(1-\omega \left(1,N_{\Omega }\left(t\right)\right)\right)+{Α}_{1}(t)\left(1-\omega \left(e^{-\rho },N_{\Omega }\left(t\right)\right)\right)+{Α}_{2}(t)\left(1-\omega \left(e^{-2\rho },N_{\Omega }\left(t\right)\right)\right), & k=0\\ {Α}_{k+2}\left(t\right)\left(1-\omega \left(e^{-\left(k+2\right)\rho },N_{\Omega }\left(t\right)\right)\right)+\sum_{c=0}^{31}\Omega_{k,c}\left(t\right)\;\alpha \left(e^{-\mathrm{k\rho}},N_{Α}\left(t\right)\right), & k=1,\ldots ,125\\ \sum_{c=0}^{31}\Omega_{k,c}\left(t\right)\;\alpha \left(e^{-\mathrm{k\rho}},N_{Α}\left(t\right)\right), & k=126,127 \end{array}\right)$$

(7) $$\Omega_{k,c}\left(t+1\right)=\left\{\begin{array}{l} {Α}_{0}\left(t\right)\;\omega (1,N_{\Omega }(t)),\\ 2\Omega_{k-1,48}\left(t\right),\\ \Omega_{k-1,c-1}\left(t\right)(1-\alpha (e^{-\left(k-1\right)\rho },N_{Α}(t))),\\ \Omega_{k-1,31}\left(t\right)(1-\alpha (e^{-\left(k-1\right)\rho },N_{Α}(t)))+{Α}_{k}(t)\;\omega (e^{-\mathrm{k\rho}},N_{\Omega }(t)),\\ \Omega_{k-1,c-1}\left(t\right),\\ 0, \end{array}\right)\;\begin{array}{l} k=0,c=32\\ k>0,c=0\\ k>0,c=1,\ldots ,31\\ k>0,c=32\\ k>0,c=33,\ldots ,48\\ \mathrm{else} \end{array}$$

### Construction and implementation of the hybrid model

In the hybrid model, the difference formulation of the ABM introduced in the last section replaces the ODE SCC. Accordingly, stem cell number *C*_*S*_ is now given by *N*_*S*_ = *N*_Α_ + *N*_Ω_ (see Eqs. (4) and (5)). Naturally, the new SCC must comply with the coupling of SCC and other compartments in the ODE model regarding cell fluxes, chemotherapy effects and feedbacks (Figure 1).

Cells passing the threshold *a*_min_ lose the ability to return to GE Α and to self-renew which means that they enter subsequent maturation stages. They constitute the efflux of the stem cell compartment $N_{S}^{\mathrm{out}}$ and the influx into the committed progenitor compartment CG. The equilibrium efflux of the ABM stem cell compartment ${\hat{N}}_{S}^{\mathrm{out}}$ relative to equilibrium stem cell number ${\hat{N}}_{S}$ is ${\hat{N}}_{S}^{\mathrm{out}}/{\hat{N}}_{S}=0.003\;h^{‒1}$ and, thus, considerably smaller than in the ODE model (${\hat{C}}_{S}^{\mathrm{out}}=a_{S}^{\mathrm{nor}}/\tau_{S}=0{.01875\;h}^{‒1}$). However, the stem cell uncertainty principle [39] makes is hard to identify stem cells and, thus, their total number precisely. On the other hand, the ODE model normalizes all cell numbers with regard to stem cell number, while the ABM uses the parameters *Ñ*_Α_ and *Ñ*_Ω_ for scaling stem cell number which have no influence on qualitative model behavior. Thus, we consider the discrepancy in output as a scaling problem and decided to match equilibrium efflux of the ABM with the one in the ODE model by using the scaling parameter *k*_*scaling*_. Therefore, in the hybrid model the efflux from S and influx to CG given by:

(8) $$\begin{array}{l} C_{S}^{\mathrm{out}}\left(t\right)=k_{\mathrm{scaling}}\frac{N_{S}^{\mathrm{out}}\left(t\right)}{N_{S}\left(t\right)\;\Delta t_{\mathrm{ABM}}}\;\text{with}\\ k_{\mathrm{scaling}}=\frac{{\hat{N}}_{S}\;{\hat{C}}_{S}^{\mathrm{out}}}{{\hat{N}}_{S}^{\mathrm{out}}} \end{array}$$

For the interval of one ABM simulation step $\left[t_{i}^{\mathrm{ABM}};t_{i}^{\mathrm{ABM}}+\Delta t^{\mathrm{ABM}}\right]$ it is assumed to be constant.

In the ODE model, chemotherapy causes a general reduction of the homogeneous stem cell population. The difference equations model distinguishes between proliferative and non-proliferative cells. Hence, it is possible to model the effect of chemotherapeutic drugs by cell-cycle specific toxicities [33]. In analogy to the ODE model, we model chemotherapy as a transient first order loss, but limited to proliferating cells in GE Ω. This is implemented by an intermediate state Ω′_*k,c*_ in each simulation step:

(9) $$\Omega_{k,c}^{'}\left(t_{n}+\mathrm{\Delta t}\right)=\left(1-k_{S}\Psi_{\mathrm{CX}}\right)\;\Omega_{k,c}\;\left(t_{n}\right),$$

where *Ψ*_*CX*_ is the characteristic chemotherapy function of the regimen considered (s. Appendix A1.1). Subsequently, all other processes such as transition between GEs, cell divisions, differentiation and regeneration are computed from this intermediate state.

Finally, feedback loops 1 and 2 of the ODE model (Figure 1, red and green) remain to be integrated into the hybrid model. Loop 1 is replaced by the intrinsic regulation of the new SCC. To implement the effect of feedback loop 2 in the hybrid model, we considered two alternative mechanisms using the number of granulopoietic cells in the bone marrow as regulator. In analogy to an increased proliferative fraction, the first one is based on an activation of quiescent stem cells by regulation of the transition probability ω. The second increases activity in the SCC by a general acceleration of all processes. Both concepts were tested extensively with various feedback functions. It turned out that this feedback loop, which is of clear importance for the ODE model, can be dropped in the hybrid model without loss of accuracy for the scenarios considered (see Discussion).

We like to emphasize here that constructing the hybrid model we did not perform any parameter fittings, i.e. all parameter values derived from the ODE and ABM model were preserved. This also applies for parameters required only for certain model scenarios such as pharmacokinetic and –dynamic parameters of G-CSF applications or chemotherapy toxicities.

### Implementation and simulations

The hybrid model was implemented in Simulink 7.4 and Matlab 7.9.0.529 (R2009b). Using the Simulink interface of user defined S-functions we implemented the difference equation formulation of the stem cell model as a Matlab S-function. Simulations of the hybrid model were carried out on SUSE Linux Enterprise Server 11 (×86 64). All parameters for the ODE model, the difference formulation of the ABM and toxicities of drugs are given in A1.2, A2.2 and A3, respectively.

## Results

A number of qualitative and quantitative simulations were performed to study the behavior of our hybrid model in comparison to the former ODE model. If not stated otherwise, we present normalized cell numbers *C*^*rel*^ in all figures.

### Qualitative model behavior

#### Depletion of the granulocyte compartment

First, we considered an initial depletion of granulocytes setting $C_{\mathrm{GRA}}=10^{-15}C_{\mathrm{GRA}}^{\mathrm{nor}}$ and initializing all other compartments with their equilibrium number *C*^*nor*^. We compared the two models regarding dynamics of granulocyte recovery. In both models, the depleted granulocyte compartment is quickly repopulated after about 8 h and equilibrates after a short overcompensation. Equilibrium is reached after about 1d. Since the loss in granulocyte numbers is signaled only via G-CSF to PGB and MGB, the feedback to the SCC is not involved. Consequently, the results of hybrid and ODE model are virtually identical (not shown).

#### Depletion of the granulocyte and maturing granulopoietic precursor compartment

**Figure 3:**
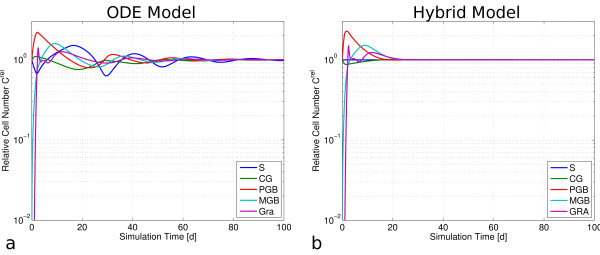
**Responses of ODE and hybrid model after depletion of compartments MGB and GRA.** In both models the depleted compartments are repopulated within 3d. **a)** In the ODE model, the feedback to the SCC causes a transient decrease in stem cell number and damped oscillations. **b)** The hybrid model does not activate the SCC and the perturbation has vanished in all compartments after ~23 days without oscillations.

Next we simulated repopulation after a depletion of the two latest stages of granulopoiesis (Figure 3). After initially setting the cell numbers in GRA and all MGB subcompartments to 10^-15^ of their equilibrium values and all remaining compartments to *C*^*nor*^, the resulting repopulation dynamics are more complex compared to the previous scenario. In the ODE model, the loss in compartment MGB is transmitted to the SCC (Figure 1, green feedback loop) and results in both, an increased proliferative fraction and a decreased self-renewal in S (s. Appendix A1.1). This induces an increased efflux and a reduction of S. In consequence, the ODE model quickly repopulates after about 3d, but the feedbacks cause damped oscillations for approximately 80 days. Without the stem cell activation via feedback 2, the hybrid model shows a very similar first response except for the stem cell compartment, but no oscillations occur.

#### Simulations of bone marrow transplantation

**Figure 4:**
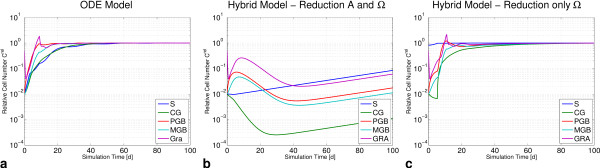
**Simulations of bone marrow transplantation with G-CSF support after myeloablative conditioning.** All bone marrow compartments were initialized with 1%, only GRA with 50% of its equilibrium value. We present relative cell numbers throughout. **a)** In the ODE model, S repopulates 50% after 18d and 100% after 60d. GRA repopulates 50% after 6d and 100% after 7d. **b)** Both GEs in the SCC of the hybrid model are reduced. Repopulation is slow and not completed after 100d. **c)** Stem cell reduction is limited to the proliferative GE Ω. S repopulates 98% in 5 days, PGB and GRA 100% after 9.5d. The other compartments repopulate within the next 40 days.

Because the differences between the models lie in the two SCC, we addressed them directly by simulations mimicking bone marrow transplantation with G-CSF support after myeloablative conditioning. For this purpose we initialized all bone marrow compartments with 1% and GRA with 50% of their equilibrium values. In all simulations G-CSF was applied until recovery of granulocyte number is achieved. In the ODE model, G-CSF was applied for 8 days. Under these conditions, the SCC regenerates 50% of its equilibrium value after 18 days and 100% after 61 days (Figure 4a). Due to the feedback mechanisms small oscillations in stem cell number occur. Repopulation of progenitor compartment CG is similar to S. The more mature compartments PGB and MGB recover faster than stem cells S and progenitors CG. Mature granulocytes GRA recover 25% of their equilibrium value after 4.6 days, 50% after 6 days and repopulate completely after 7 days. If G-CSF application is extended to 20 days, recovery dynamics does not change in GRA, but clearly in the SCC despite of no direct effects of G-CSF on S. Regeneration in the SCC is accelerated considerably, repopulating 50% after 13.5 days and 100% after only 19 days (data not shown).

Analogously, we tested the performance of the hybrid model in this scenario (Figure 4b). In both GEs Α and Ω of the SCC the content was reduced to 1% of its equilibrium value. Here, recovery is very slow compared to the ODE model and not completed after 100d. Initially, only GRA and CG decrease clearly. Stem cell number S decreases only slightly, because the cells found at low affinities *a* in GE Ω rarely switch to Α, but rather leave the SCC. After these cells have left the SCC, the intrinsic regulation of the SCC first repopulates S before it generates progenitor cells again. Initially maximal amplification allows cell numbers in PGB, MGB and GRA to grow. Then the efflux of progenitor cells from S drops to only approximately 0.05% of its equilibrium value and causes a decline of all subsequent cell numbers. The nadir propagates through all compartments eventually reaching GRA, which starts to regenerate at d45.

If only Ω is reduced in the SCC, 82% of the stem cells remain in GE Α, repopulate GE Ω and restore the efflux of progenitors (Figure 4c). Reduced transit times in later departments and increased amplification lead to a repopulation of GRA after only 10 days.

Hence, fundamentally different behavior arose from those three scenarios. While recovery is fast in the ODE model, in the hybrid model it is not achieved within a reasonable time frame, if all stem cells are reduced. If only GE Ω is affected, maximal reduction of GRA is similar to the ODE model, but stem cell dynamics are largely different.

Preliminary adaptations of the hybrid model to this scenario were motivated by the effect of myeloablation on the bone marrow environment (s. Discussion). We altered the transition characteristics of the SCC that model limited capacities of both GEs. Without changing their values at the equilibrium cell numbers, the feedback was made more sensitive by increasing the sensitivity of the sigmoidal transition characteristics to cell number (s. Appendix A2.2). This resulted in much faster repopulation dynamics after reduction of both GEs (s. Appendix, Figure 9). Higher values of the transition characteristics at small cell numbers resulted in stem cell repopulation after 31 days and recovery of 25% of granulocytes after 36 days. In these simulations the fast repopulation dynamics of the stem cell compartment results in oscillations, too. However, we have not elaborated this approach for application to bone marrow transplants, because here we limit ourselves to the combination of the two models as a proof of principle.

#### Chronic irradiation

After single initial reductions we were interested in the behavior of the system when exposed to a continuous damage as in the case of chronic irradiation. In mice irradiated with a daily dose of up to 0.6 Gy/d this continuous damage results in a strong, dose-dependent decline of CFU-S numbers during the first days of irradiation. Then CFU-S numbers were found to stabilize at a lower level (see [15] and references therein). Cell numbers of later cell stages also decrease in a dose-dependent way, but until a dose of about 0.6 Gy/d their cell numbers are less sensitive compared to CFU-S. Then they decline rapidly and the animal dies. These results suggest that until a certain threshold of damage, the system can compensate the decline of CFU-S. We expect a similar behavior for human hematopoiesis and checked whether the hybrid model can reproduce this behavior, too.

**Figure 5:**
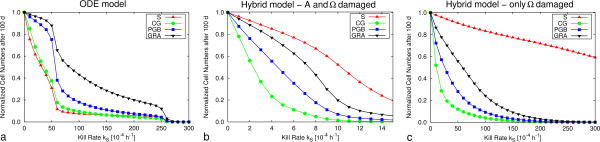
**Simulations of chronic irradiation.** Constant kill rates are applied to S, CG and PGB (with *k*_*CG*_ = *k*_*PGB*_ = 0.66 *k*_*S*_). Cell numbers in S, CG, PGB and GRA are recorded after 100 days. **a)** In the ODE model reductions in S and CG are similar. PGB and GRA almost maintain cell numbers at low doses. At higher doses cell numbers in PGB and GRA decrease rapidly. **b,c)** In the hybrid model damage in S is smaller than in all other compartments. **b)** If both GEs Α and Ω are damaged, the system is sensitive to kill rates that are an order of magnitude smaller than for the ODE model. **c)** If damage is restricted to GE Ω, the SCC is more robust at kill rates comparable to those of the ODE model. All other compartments are more sensitive in comparison to stem cells.

Following Wichmann and Loeffler, chronic irradiation is modeled as a constant first order kill with rates *k*_S_ in S and *k*_CG_ = *k*_PGB_ = 0.66 *k*_S_ in CG and PGB [15]. In the hybrid model, we consider applications of the kill rate *k*_S_ to either both GEs or only to the proliferative GE Ω. For each scenario, we recorded the contents of cell compartments after 100d. In the ODE model, S and CG show the same dose dependence (Figure 5a). At small doses, their cell numbers are highly sensitive to small dose-increments. At higher doses the SCC responds to the stem cell loss by higher proliferation and self-renewal and stabilizes at low stem cell numbers until the system dies for kill rates *k*_S_ > 0.025 h^-1^. At stem cell kill rates lower than 0.005 h^-1^ the rapid loss of S and CG is compensated by increased proliferative fractions and amplifications resulting in only slightly reduced cell counts of circulating granulocytes (GRA). For doses of 0.005 h^-1^ < *k*_S_ < 0.025 h^-1^ the loss can no longer compensated and GRA starts to decline rapidly.

In contrast, in the hybrid model reduction in S is smaller than in all other compartments and the greatest cell loss is always found in CG (Figure 5b,c). During the differentiation process the amplifying compartments compensate this loss to some extent, too. If both GEs are damaged, the system is more sensitive to the chronic damage and collapses at kill rates an order of magnitude smaller than the ODE model (Figure 5b). At a kill rate *k*_S_ ~ 10^-3^ h^-1^ proliferation in GE Ω fails to counterbalance the losses in both GEs, the SCC cannot stabilize anymore and dies. If only GE Ω is affected, S itself is very robust against irradiation, but the kill in GE Ω minimizes progenitor efflux. Yet at intermediate kill rates, this strong reduction of efflux cannot be compensated at later cell stages (Figure 5c).

### Quantitative modeling - application to chemotherapy data

After studying the behavior of the hybrid model qualitatively in comparison to the ODE model, we now apply it quantitatively to clinical data provided by the NHL-B trial of the German High Grade Non-Hodgkin-Lymphoma Study Group [40,41]. Since one of us (M. Loeffler) is the responsible biostatistician of this trial group, we have access to raw patient data for our modeling purposes. Leukocyte counts were determined in patients receiving one of four CHOP-like multicycle polychemotherapy regimens. The schedules differ in application of etoposide (dose-intensification) and duration of therapy cycle (time-intensification). The time-intensified regimens require G-CSF support. For comparison with the normalized simulation results leukocyte number is assumed to be proportional to granulocyte number with normal value of 7000 leukocytes per μl blood [16]. Details of therapies can be found in the Appendix, Table 5. The pooled patient data is available online as supplemental data (s. Appendix: Additional file 1).

#### Chemotherapy regimen without G-CSF

**Figure 6:**
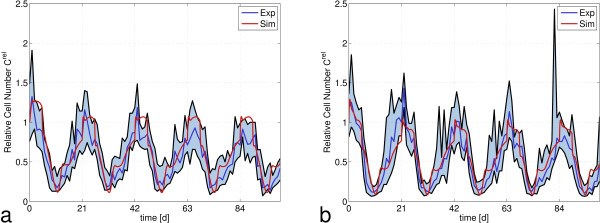
**Comparison of the predictions of the hybrid model with clinical data on leukocyte dynamics in peripheral blood during chemotherapy without G-CSF.** Clinical data of **a)** CHOP-21 and **b)** CHOEP-21 administration (Blue: median of patients, black: 25 and 75 percentiles) are compared with corresponding simulation results of the hybrid model (red). Simulation results fit well to clinical data in the sense that they lie in the interquartile range of data for almost all time points. Cell numbers are normalized with respect to the average WBC/leukocyte count value of healthy individuals (7000 cells/μl).

The hybrid model distinguishes between proliferative and quiescent stem cells. For the mitosis-related effect of the applied drugs we restrict their toxic effect on proliferating stem cells. The effect of the three cytotoxic drugs applied in the CHOP regimen is modeled by a single set of toxicity parameters, one parameter for each compartment Ω, CG, PGB and MGB. Toxicity parameters were taken from [17]. In particular, we used the same parameter value for toxicity in Ω as for our former stem cell toxicity. The effect of prednisone is modeled as a prolongation of granulocyte half-life according to Bishop et al. [42] and Dale et al. [43]. The results of the simulation of CHOP-21 in comparison to clinical data are shown in Figure 6a. After an initial increase of leukocytes due to prednisone application, the cell counts decrease to about 10% of their normal value until day 11, followed by a recovery phase until the start of the next cycle. Model results fit well to the clinical data in the sense that the model prediction is in the interquartile range of the clinical data for almost all time points. For the CHOEP regimen, an additional set of toxicity parameters representing the toxicity of the drug etoposide is used. Again, model predictions fit well to clinical data applying the same toxicity parameters as used in Scholz et al. [17] (Figure 6b).

#### Chemotherapy regimen with G-CSF

**Figure 7:**
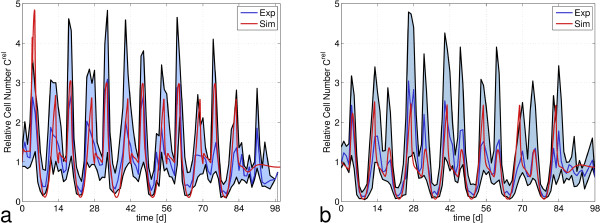
**Comparison of the predictions of the hybrid model with clinical data on leukocyte dynamics in peripheral blood during chemotherapy with G-CSF support.** Clinical data of **a)** CHOP-14 and **b)** CHOEP-14 administration (Blue: median of patients, black: 25 and 75 percentiles) are compared with corresponding simulation results of the hybrid model (red). The effect of growth factor support is reflected by the peak approximately one day after starting the G-CSF treatment at day 4 in each cycle. As for the regimens without G-CSF, simulation results fit well to clinical data. Cell numbers are normalized with respect to the average WBC/leukocyte count value of healthy individuals (7000 cells/μl).

The CHOP-14 and CHOEP-14 regimen use the same schedule of drug administration, but reduce regeneration time. The damage caused by this intensification is counterbalanced by application of the growth factor G-CSF between day 4 and day 13 of each cycle. Therefore we have used the same toxicity parameters as for a cycle duration of 21 days. G-CSF applications are covered by the pharmacokinetic and -dynamic model of G-CSF provided by the ODE model and given in the Appendix A1.1 [17]. Again, for both scenarios a good agreement of hybrid model simulations and data was found for the original toxicity parameters and without other adaptations (Figure 7). Even though a completely different model of stem cell organization and regulation was used in the hybrid model, the resulting leukocyte dynamics under chemotherapy is consistent with both, the ODE model and the clinical data.

#### Comparison of stem cell dynamics during chemotherapy

**Figure 8:**
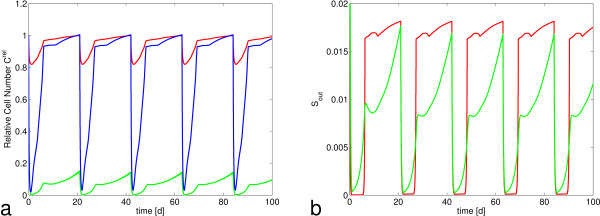
**Comparison of stem cell dynamics in ODE and hybrid model. a)** Stem cell number in the ODE model (green) is heavily reduced and does not recover within one cycle. In the hybrid model, total stem cell number (red) is only reduced to ~80%. Depletion of proliferative stem cells (blue) is similar to the ODE model, but regeneration is fast. **b)** Effluxes from the ODE SCC (green) and the hybrid model (red) almost vanish after drug administration, but increase simultaneously around day 7.

To explain this phenomenon, we had a closer look at the behavior of both SCCs and their effluxes in the course of CHOP-21 simulations (Figure 8). During CHOP-21 application, total stem cell numbers in ODE and hybrid model are reduced (Figure 8a). But while the ODE SCC is almost eliminated, the SCC of the hybrid model maintains the quiescent population and, thus, more than 80% of its total number due to the limitation of cytotoxicity to proliferating cells. Although proliferative cells are equally reduced in both models, in the hybrid model the quiescent cells quickly repopulate the proliferative GE after the cytotoxic effect of drug administration has passed, and facilitate a nearly complete recovery in each cycle. In contrast, the SCC of the ODE model has to regrow from only 0.23% of its equilibrium population. Therefore it maximizes its proliferative fraction *a*_*S*_ and puts all cells to proliferation, but even so it only restores about 20% of its equilibrium population until the beginning of the next cycle.

Despite the huge differences in the dynamics of stem cell numbers after chemotherapy, the effluxes of both SCC show similar dynamics (Figure 8b). In the beginning of each cycle both almost vanish and around day 7 both increase rapidly to more than 50% of their equilibrium value. At this time efflux is almost restored in the hybrid model, because GE Ω recovered almost completely. In the ODE model the feedback loops regulating proliferative fraction *a*_*S*_ and probability of self-renewal *p* allow an increase of efflux to about 50% of its equilibrium value although stem cell number *C*_*S*_ is still around 10% of its equilibrium value $C_{S}^{\mathrm{rel}}$. In consequence, on the level of mature granulocytes, both models agree with each other and the clinical data as well, because the differences in efflux from the stem cell compartment are sufficiently small to be compensated by the feedback controlled amplification in subsequent bone marrow compartments.

## Discussion

The number of attempts to model hematopoiesis or hematopoietic sub-processes is considerable (e.g. [8-12,16-18,44-49]). Usually established models are extended to cover new scenarios [45,46,49-51]. However, despite the often complementary scenarios covered by yet existing models, little effort was made to unite them in order to show their compatibility/consistency, to seek confirmation or to address open questions. Here, we pursue such a cross-validation of two established models for hematopoiesis developed in different contexts and on the basis of different experimental data by combining them into one comprehensive hybrid model for human granulopoiesis. The two models were chosen for their range of clinically relevant applications, established parameter sets, in particular for human hematopoiesis, and their complementary level of description. Rather than constructing a model from scratch, we rely on these complementary modeling works by adopting all their model assumptions, equations and parameter settings. Therefore only adaptations necessary to combine them were made. Due to the different contexts in which the models were developed, it was by far not clear whether such an attempt will be successful. Our approach can therefore be understood as a second level of modeling by combining established models in order to construct more comprehensive ones. To solve this task, we examined different hypotheses for model combination and performed a number of qualitative and quantitative benchmark simulations to compare the hybrid model with the established ODE model, to test whether the combined models are compatible and to check whether the new hybrid model has potential to explain a broader range of scenarios.

The ordinary differential equation-based lineage model for granulopoiesis developed by Scholz et al. [17] served as the backbone of our hybrid model. This model describes the dynamics of cell stages from stem cells to circulating granulocytes and the granulopoietic growth factors G-CSF and GM-CSF. It succeeded in modeling and prediction of a number of clinically relevant scenarios of chemotherapy and G-CSF applications [17]. The model of chemotherapy is based on transient cell depletions of bone marrow compartments in dependence on drugs and drug doses. Alternative approaches to model chemotherapy are based on assumptions regarding increased apoptosis and impaired proliferation. We relied on our approach since our former modeling proved to be successful in explaining clinical data sets of different chemotherapies [16,17,19].

Because stem cells are represented as a homogenous population, modeling of processes influenced by stem cell heterogeneity such as cell cycle dependent drug toxicity is difficult in the ODE framework. This was motivation for us to combine it with a more detailed model of hematopoietic stem cells which was developed to understand e.g. clonal competition and immune-chemotherapy effects in leukemia treatment [27,28,32]. Combination is realized by replacing the stem cell compartment of the ODE model by a difference equation formulation of the agent-based model of HSC organization [34].

Most of the hematopoietic models published so far do not provide a detailed view on the stem cell level, since they either do not model stem cell dynamics [8-10,14,48] or treat them similarly simple as the ODE model [11,12]. We chose the ABM for HSC introduced by Roeder and Loeffler [26] to replace the stem cell compartment of our ODE for two reasons: it represents the stem cell population in a highly structured way (two niches, affinity, cell cycle) and has been proven successful in a broad range of scenarios relevant for clinical applications such as Imatinib-treatment of chronic myeloid leukemia in humans [30,32]. Replacement is performed on the basis of a difference equations formulation of the ABM proposed by Kim et al. [34]. Because the difference formulation conserves the ideas and behavior of the ABM, combination of these different types of models (ABM, ODE) at different scales (single cell, cell populations) is also interesting from a general systems-biological point of view. The stem cell models of Adimy [44] and Mackey [13] describe the stem cell population with less detail, but are continuous alternatives for substitution of the stem cell compartment of our ODE model. We have also experimented with ODE compartment models of proliferating and dormant stem cells in the framework of a murine model of haematopoiesis under chemotherapy with fairly good results (unpublished).

For similar computation times in both sub-models, we chose to take advantage of two simplifications of the ABM: (i) a difference formulation, which does not deteriorate accuracy [34] and (ii) the approximation of the probabilistic transitions between GEs by their expectation values. The first simplification impedes the analysis of individual cell fates and clonal contributions, but separate matrices and parameter sets allow modeling of limited numbers of stem cell subpopulations, such as leukemic and normal cells. The second makes the model deterministic and results in continuous cell numbers, but eliminates stochastic variation among realizations that could be interpreted as variation among individuals. However, if desired, stochastic transitions can be easily re-established by using binomially distributed random numbers.

A major challenge of our approach was to substitute the feedback-loop signaling from more mature bone marrow compartments back to stem cells. In the ODE model this and the internal stem cell feedback control proliferative fraction (*a*_S_) and self-renewal probability (*p*) in the SCC (s. Appendix A1.1). Because the ABM provides an intrinsic regulation of proliferation and stem cell maintenance, the stem cell feedback is replaced by this internal regulation. To translate the feedback of the bone marrow to stem cells into the hybrid model, we re-interpreted the stem cell parameters *a*_S_ and *p* of the ODE model in terms of the new stem cell model. In the ABM the proliferative fraction is given by the fraction *N*_Ω_/( *N*_Ω+_*N*_Α_) of stem cells in GE Ω and is regulated by the transitions between the GEs. These in turn are controlled by the transition intensities α and ω. Accounting for experimental results on activation of dormant HSC after G-CSF stimulation [38], we first tested a dependence of the probability ω for a transition Α → Ω on the number of granulopoietic cells in the bone marrow. However, simulations showed a long delay between stem cell activation and increase of progenitor efflux. This delay led to overcompensation at the level of granulocytes and, thus, a clear deviation from the clinical data. Alternatively we considered stem cell activation as an acceleration of all processes in the ABM SCC. The resulting shorter cell cycle and faster differentiation leads to an increase in cell divisions in GE Ω and also progenitor efflux from GE Ω. In combination with the regulation of transition intensity ω, it reduces the overcompensation mentioned above, but it does not eliminate it. Independent of this combination, it shifts the granulocyte nadir after chemotherapy by an earlier increase in production of progenitor cells. This causes additional deviation from the clinical data. Both, regulation of ω and acceleration, were implemented as continuous piecewise functions of the bone marrow cellularity G. In the sensitive range around equilibrium conditions we tested linear and quadratic dependences on bone marrow cellularity G. Outside this range we assumed constant minimal and maximal values. Various ranges and sensitivities of both feedback functions and their combinations were tested, but it turned out that the hybrid model agrees best with the quantitative data after chemotherapy if the native stem cell regulation of the ABM is used without an additional feedback. Therefore, we decided to perform all simulations only with the original stem cell regulation of the ABM.

Except for this elimination of a feedback and the restriction of the effect of chemotherapy to proliferating cells, we did not introduce any modifications of both sub-models, in particular with respect to parameter settings. However, this substitution altered the number of model parameters. With the ODE stem cell compartment we eliminated eight parameters of the former ODE model including those of loop 2, but introduced 16 parameters with the ABM model. Because these parameters were neither fitted nor adapted, we added no additional degrees of freedom to achieve a better fit to the data. A fine tuning is possible, but contradicts the idea of combining the models as a proof of compatibility and confirmation. It might be necessary when applying the hybrid model to new and more complex scenarios not yet covered by both submodels, e.g. more complex chemotherapies, diseased granulopoiesis and bone-marrow transplantation (s. Section “Simulations of bone marrow transplantation” and A4), which is planned for the future.

In our qualitative simulations, we observed that after damaging mature compartments, the recovery of circulating cells is similar under the former ODE model and the new hybrid model. But at early cell stages, the ODE model shows damped oscillations which do not occur for the hybrid model. Lack of data prevents us to decide which behavior is in better agreement with biological reality.

**Figure 9:**
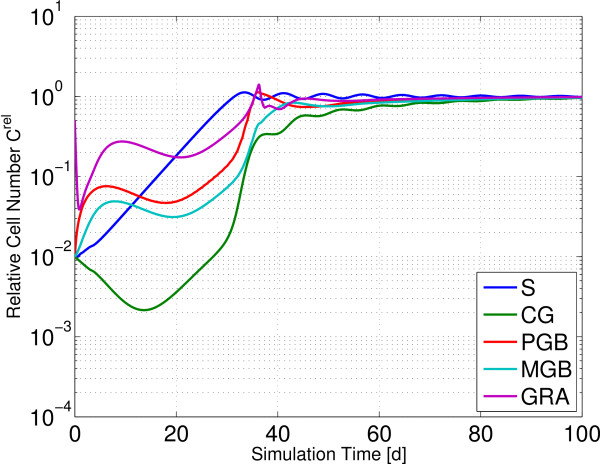
**Preliminary simulation of myeloablative bone marrow transplant with modified transition characteristics.** While keeping the equilibrium values of the transition characteristics nearly unchanged, for smaller cell numbers N_Α_ and N_Ω_ the transition characteristics increase much stronger than for the established human parameter set. The more dynamic switching behavior results effectively in stem cell activation and much faster recovery. Stem cells repopulate completely after 31 days and GRA recovers 25% of its equilibrium value after 36 days. Again G-CSF application was continued until the recovery of GRA.

Main differences between the hybrid and the ODE model arose after perturbations of the stem cell pool itself. In our ODE model simulations of myeloablative bone marrow transplants, the SCC repopulates completely within approximately 50 days and GRA recovers its equilibrium value after only 7 days. This is well below the lower boundary of the wide range of recovery times for absolute neutrophil count after bone marrow transplantation [52,53]. While repopulation in the ODE model is too fast, in the hybrid model it is far too slow. Only if damage in the SCC is restricted to proliferating cells, reconstitution is completed within reasonable times. However, the latter is not comparable to myeloablation since about 82% of the stem cells remain unaffected. Myeloablation very likely triggers a wide destruction of bone marrow structure and the niche environment as is known for irradiation in mice [54-56]. Hence, our model does not cover this scenario so far. It would require adaptations of the model mechanisms that consider changes in environment and signaling context. One approach could be a stronger dependence of transitions on the capacity of the damaged bone marrow niche after conditioning and, consequently, a higher fraction of proliferative cells until the niche is restored. In preliminary simulations we used different transition characteristics with higher values for low cell numbers without changes for equilibrium numbers (s. Appendix A4, Figure 9). Consequently, cells are more likely to switch GEs, which can be interpreted as a reduced ability of each GE to keep HSC in a stable state. Driven by the instable quiescence the stem cells enabled a recovery of 25% of granulocyte number within 36 days. This is close to the upper boundary found in clinical studies [52,53] and demonstrates the potential of the hybrid model for applications to high-dose chemotherapy and stem cell transplantation. The transition characteristics of the new stem cell compartment control the activation of stem cells and capacity of the two GEs. They also determine the ratio of dormant and active HSC and their rate of exchange. Hence, a detailed elaboration on changing growth environments and its application to high-dose chemotherapy is possible, but goes beyond the scope of this article.

Similar arguments hold for our qualitative simulations of chronic irradiation. But here neither a relation of experimental irradiation dose and model toxicity parameters nor a clear matching of model compartments and CFU-S exists, so our results can only be interpreted in a qualitative way. The compensation of stem cell loss by regulated amplification in more committed compartments observed for radiation experiments in mouse [15] is conserved in the hybrid model if one identifies CFU-S with the compartment CG.

Despite the differences in stem cell dynamics for bone marrow transplantation and chronic irradiation, quantitative application of the hybrid model to clinical data of four scenarios of conventional chemotherapies with and without G-CSF treatment was successful. Here, we restricted the damaging effect of chemotherapy to the proliferating stem cell sub-compartment of the hybrid model and applied the stem cell kill rates estimated for the former ODE model. This biologically more plausible approach of cell cycle specific chemotherapeutic drugs in the hybrid model avoids the apparently unrealistic reduction that is predicted by the ODE model [17,33]. We chose the CHOP-like scenarios of the NHL-trial, because they can serve as benchmark scenarios for the modeling of general, more complex chemotherapies [17,19]. Agreement of hybrid model and data was good for all chemotherapy regimens without any adaptation of parameters and only the minute change of model structure that were necessary for the combination of the submodels.

The decision to drop the feedback of bone marrow cellularity to stem cells was based on the comparison with quantitative data of the CHOP-like chemotherapy regimens. These regimens intend to maximally affect tumors at the lowest toxicity for normal tissues. Hence, it is conceivable that under these therapies, the bone marrow environment remains widely intact. Our results therefore suggest that HSCs, in particular the dormant subpopulation, represent an important population that is highly protected and only activated after more severe damage than done by these four damage-optimized regimens. Activation of dormant HSCs only after heavy damage also explains why diseases involving HSCs require myeloablative therapies. For such strong damage, our preliminary model simulations suggest a reduced stability and activation of the dormant state due to this environmental destruction, which is reflected in transition characteristics that are more sensitive to bone marrow cellularity.

The reason for the agreement of the two models for the four CHOP-like regimens is the similarity of efflux dynamics of the two SCC. Both models agree on a proliferative fraction of about 15% of the stem cells at equilibrium, too, but assume different cycle times (8 h in the ODE model, 48 h in the ABM). Proliferative activity in the stem cell compartment is computed from proliferative fraction and cycle time. In both models, it covers a similar range, but follows opposite dynamics during chemotherapy. The ODE SCC activates all stem cells in order to regenerate the nearly eliminated SCC and sends them into proliferation (*a*_S_ = 1), but is unable to recover within one chemotherapy cycle. In contrast, the hybrid model predicts also a nearly elimination of proliferating HSC (*a*_S_ ~ 0) by the cytotoxic drugs, but quickly repopulates them from quiescent cells without activating more HSC than in equilibrium. Hence, the two model scenarios suggest contrary hidden dynamics, but both explain the clinical data equally well.

To evaluate which model better resembles biology in the situation at hand, measurements of stem cell numbers during chemotherapy would be required, which is impossible for humans. Even if biopsies would be available for analysis, the problem of identifying quiescent and proliferative HSC subpopulations and their cycle times remains. Generally, the question of the ratio of quiescent and active stem cells and their switching frequency is heavily discussed [38,57]. In the present situation, there are various estimates, but mainly for mouse HSC which seem to differ in many aspects from the human system [58,59]. Hence, for the better availability of experimental data, an adaptation of the model concept to the murine system is planned for the future. The advantage of the hybrid model to distinguish between the two populations of quiescent and proliferating stem cells becomes more powerful for murine hematopoiesis and related experimental possibilities. Here it provides a well suited tool to study the equilibrium of dormant/active HSC and its impact on hematopoiesis. Insights from mouse modeling could later improve our modeling in humans. This could result in a more universal model of human granulopoiesis, whose clinical applications could go beyond existing models. Additionally, the hybrid model will serve as the basis for a comprehensive model of complete hematopoiesis by integrating the model of stem cell lineage commitment and the other models of hematopoietic lineages developed by our group since they rely on the same stem cell model [15,17,18,47].

## Conclusions

In our hybrid model we have successfully combined two complementary models for stem cell organization and granulopoiesis by replacing the former stem cell compartment of the lineage model and corresponding regulations. The hybrid model features a novel combination of both, a detailed representation of stem cells and the description of later cell stages of granulopoiesis including the effect of growth factor signaling. In particular, its representation of stem cell states allows the modeling of cell cycle dependent cytotoxic effects of chemotherapy. A number of qualitative and quantitative simulations of the hybrid model revealed very different behaviors of the former and the new stem cell model. Nevertheless, quantitative application of the hybrid model to four CHOP-like chemotherapy regimens showed that the hybrid model explains the clinically observed dynamics of leukocyte numbers equally well. Model validation would require more detailed experimental data on human HSC.

This novel combination paves the way for studying not yet addressed scenarios such as high-dose chemotherapy, radiotherapy and diseased hematopoiesis. It serves as another step towards a comprehensive model of hematopoiesis comprising models of stem cell regulation, lineage commitment, bone marrow cell populations, growth-factors and chemotherapy actions.

## Appendix

### A1. Details of the ODE model

#### A1.1 Model equations

##### The regulatory Z-function

 This regulatory function is used to calculate amplification *A* in CG, PGB and sub-compartment G6 in MGB as well as the transit time in MGB. It also determines the endogenous production of the growth factors.

$$Z_{Y}\left(C_{X}^{\mathrm{rel}}\right)=Y^{\max}-\left(Y^{\max}-Y^{\min}\right)\exp\left(-\ln\left(\frac{Y^{\max}-Y^{\min}}{Y^{\max}-Y^{\mathrm{nor}}}\right)\right){\left(C_{X}^{\mathrm{rel}}\right)}^{b_{Y}}$$

This representation allows regulating the quantity $Y=Z\left(C_{x}^{\mathrm{rel}}\right)$ between a minimum and a maximum in dependence on $C_{x}^{\mathrm{rel}}$. In steady-state $\left(C_{x}^{\mathrm{rel}}=1\right)$ it is equal to *Y*^nor^*.* The parameter *b*_*Y*_ allows choosing different degrees of sensitivity. Details can be found in [17].

#### Characteristic function of chemotherapy

Chemotherapy is modeled in all compartments as a transient depletion of cells following a first order kinetics. The rate of this kinetics is given as the product of a kill rate *k*_*X*_ (drug and compartment specific) and a characteristic function Ψ_CX_ modeling its time dependence:

$$\begin{array}{cc} \Psi_{\mathrm{CX}}\left(t\right)=\left\{\begin{array}{l} f_{\mathrm{fc}}\\ 1\\ 0 \end{array}\right) & \begin{array}{l} \;\mathrm{for}\;t_{1}<t\leq t_{1}+1d\\ \;\mathrm{for}\;t_{i}<t\leq t_{i}+1d\\ \mathrm{otherwise}, \end{array} \end{array}$$

where *t*_*i*_ are the time points of chemotherapy application. If drugs are applied at different schedules, the different chemotherapy functions are added.

#### Probability of self-renewal in S

The probability of self-renewal in the stem cell compartment S is controlled by numbers of stem and granulopoietic cells in the bone marrow:

$$p=p_{\delta }\tanh\left(-\vartheta_{S}\left(C_{S}^{\mathrm{rel}}\left(t\right)-1\right)-\vartheta_{G}\left(C_{G}^{\mathrm{rel}}\left(t\right)-1\right)\right)+0.5.$$

$$\begin{array}{cc} \vartheta_{S}=\left\{\begin{array}{l} \frac{2}{C_{S}^{\mathrm{rel}}{\left(t\right)}^{0.6}}\\ 2 \end{array}\right) & \begin{array}{l} \mathrm{for}\;C_{S}^{\mathrm{rel}}\leq 1\\ \mathrm{for}\;C_{S}^{\mathrm{rel}}>1 \end{array} \end{array}$$

The representation of *p* by tanh was used to regulate the quantity symmetrically between a minimum and a maximum and to guarantee maximum sensitivity in steady-state. See Wichmann/Loeffler for further details [34].

#### Proliferative fraction *a*_*X*_ in compartments S and CG

Also the proliferative fraction *a*_*X*_ in the compartments S and CG is regulated by the numbers of stem and granulopoietic cells in the bone marrow:

$$x=\omega_{G\;}\mathrm{In}\;C_{G}^{\mathrm{rel}}\;\left(t\right)+\omega_{S}\cdot \left\{\begin{array}{l} \begin{array}{l} \mathrm{In}\;C_{S}^{\mathrm{rel}}\left(t\right) \end{array}\\ C_{S}^{\mathrm{rel}}\;\left(t\right)-1 \end{array}\right)\;\begin{array}{l} \mathrm{for}\;C_{S}^{\mathrm{rel}}\leq 1\\ C_{S}^{\mathrm{rel}}>1 \end{array}$$

$$\begin{array}{ll} y= & \frac{1}{2\ln2}\left(\ln\left(\frac{a_{X}^{\mathrm{int}}-a_{X}^{\max}}{a_{X}^{\min}-a_{X}^{\mathrm{int}}}\right)-\ln\left(\frac{a_{X}^{\mathrm{nor}}-a_{X}^{\max}}{a_{X}^{\min}-a_{X}^{\mathrm{nor}}}\right)\right)x\\ & +\frac{1}{2}\ln\left(\frac{a_{X}^{\mathrm{nor}}-a_{X}^{\max}}{a_{X}^{\min}-a_{X}^{\mathrm{nor}}}\right) \end{array}$$

$$a_{X}=\frac{a_{X}^{\max}e^{-y}+a_{X}^{\min}e^{y}}{e^{-y}+e^{y}}$$

This regulation was also adopted from Wichmann/Loeffler p.65 [15] and Scholz et al. [17] respectively. In compartment *X* (=S or CG) the proliferative fraction *a*_*X*_ is regulated between a minimum and a maximum value, $a_{X}^{\min}$ and $a_{X}^{\max}$. The quantity *x* represents the weighted logarithmic relative size of the regulating compartments (S and G). The normal value $a_{X}^{\mathrm{nor}}$ and the value for intensified stimulation $a_{X}^{\mathrm{int}}$ corresponding to *x* = −ln2 are fixed parameters, too.

#### Amplification splitting in all proliferating compartments

According to Scholz et al. amplification is always divided into an amplification of the influx (“in”) and the efflux (“out”) respectively (see [17] for further details).

#### Stem cell compartment S

$$\begin{array}{l} \frac{d}{\mathrm{dt}}C_{S}=\left(2p-1\right)C_{S}\frac{a_{S}}{\tau_{S}}-k_{S}\psi_{\mathrm{CX}}C_{S}\\ C_{S}^{\mathrm{out}}=2\left(1-p\right)C_{S}\frac{a_{S}}{\tau_{S}} \end{array}$$

#### Granulopoietic progenitor cells CG

$$\begin{array}{l} A_{\mathrm{CG}}=Z_{A_{\mathrm{CG}}}\left(C_{\mathrm{GM}-\mathrm{CSF}}^{\mathrm{rel}}\right)\\ \frac{d}{\mathrm{dt}}C_{\mathrm{CG}}=C_{S}^{\mathrm{out}}A_{\mathrm{CG}}^{\mathrm{in}}-C_{\mathrm{CG}}\frac{a_{\mathrm{CG}}}{T_{\mathrm{CG}}}-k_{\mathrm{CG}}\Psi_{\mathrm{CX}}C_{\mathrm{CG}}\\ C_{\mathrm{CG}}^{\mathrm{out}}=C_{\mathrm{CG}}A_{\mathrm{CG}}^{\mathrm{out}}\frac{a_{\mathrm{CG}}}{T_{\mathrm{CG}}} \end{array}$$

#### Proliferating granulopoietic precursor cells PGB

$$A_{\mathrm{PGB}}=Z_{A_{\mathrm{PGB}}}\left(C_{G-\mathrm{CSF}}^{\mathrm{rel}}\right)$$

$$\frac{d}{\mathrm{dt}}C_{\mathrm{PGB}}=C_{\mathrm{CG}}^{\mathrm{out}}A_{\mathrm{PGB}}^{\mathrm{in}}-C_{\mathrm{PGB}}\frac{a_{\mathrm{PGB}}}{T_{\mathrm{PGB}}}-k_{\mathrm{PGB}}\Psi_{\mathrm{CX}}C_{\mathrm{PGB}}$$

$$C_{\mathrm{PGB}}^{\mathrm{out}}=C_{\mathrm{PGB}}A_{\mathrm{PGB}}^{\mathrm{out}}\frac{a_{\mathrm{PGB}}}{T_{\mathrm{PGB}}}$$

#### Maturing granulopoietic precursor cells MGB G4-6

To guarantee distributed transit times, the compartments G4-6 are further subdivided into *N*_*G4*_*, N*_*G5*_ and *N*_*G6*_ subcompartments (for details see [17]). While the maturing time is regulated in all subcompartments, amplification is regulated only in G6 (*A* < 1) which reflects post-mitotic apoptosis, while *A* = 1 in G4/G5. Below the equation for G4 are given. The corresponding equations for G5/G6 are obtained by replacing G4 with G5/G6 and PBG with G4/G5, respectively.

$$\begin{array}{l} T_{G4}=Z_{T_{G4}}\left(C_{G-\mathrm{CSF}}^{\mathrm{rel}}\right)\\ \mathrm{Regulation}\;\mathrm{only}\;\mathrm{in}\;G6:A_{G6}=Z_{A_{G6}}\left(C_{G-\mathrm{CSF}}^{\mathrm{rel}}\right)\\ C_{G4}=\sum_{i=1}^{N_{G4}}C_{G4\_i}\\ \frac{d}{\mathrm{dt}}C_{G4\_1}=C_{\mathrm{PGB}}^{\mathrm{out}}-C_{G4\_1}\frac{N_{G4}}{T_{G4}}-k_{\mathrm{MGB}}\Psi_{\mathrm{CX}}C_{G4\_1}\\ \begin{array}{cc} \frac{d}{\mathrm{dt}}C_{G4\_i}=C_{G4\_\left(i-1\right)}^{\mathrm{out}}-C_{G4\_i}\frac{N_{G4}}{T_{G4}}-k_{\mathrm{MGB}}\Psi_{\mathrm{CX}}C_{G4\_i} & \mathrm{for}\;i=2,\ldots ,N_{G4} \end{array}\\ \begin{array}{cc} C_{G4\_i}^{\mathrm{out}}=A_{G4\_i}C_{G4\_i}\frac{N_{G4}}{T_{G4}} & \mathrm{for}\;i=1,\ldots ,N_{G4} \end{array}\\ C_{G4}^{\mathrm{out}}=C_{G4\_N_{G4}}^{\mathrm{out}} \end{array}$$

#### Granulocytes GRA

$$\begin{array}{l} \frac{d}{\mathrm{dt}}C_{\mathrm{GRA}}=C_{\mathrm{MGB}}^{\mathrm{out}}-\frac{C_{\mathrm{GRA}}}{T_{\mathrm{GRA}}}\\ T_{\mathrm{GRA}}=T_{\mathrm{GRA}}^{\mathrm{nor}}\left(1+T_{\mathrm{GRA}}^{\mathrm{Pr}\mathrm{ed}}\;\Psi_{\mathrm{Pr}\mathrm{ed}}\right) \end{array}$$

Ψ_*Pred*_ is the characteristic function of prednisone administration analogous to the characteristic function of chemotherapy (A2) but without first cycle effect (i.e. *f*_*fc*_ = 1).

#### Granulocyte-Macrophage Colony Stimulating Factor GM-CSF

$$\begin{array}{l} \;P_{\mathrm{GM}-\mathrm{CSF}}^{\mathrm{endo}}=Z_{\mathrm{GM}-\mathrm{CSF}}\\ \times \left(\frac{\omega_{\mathrm{CG}}C_{\mathrm{CG}}+\omega_{\mathrm{PGB}}C_{\mathrm{PGB}}+\omega_{G4}C_{G4}+\omega_{G5}C_{G5}+\omega_{G6}C_{G6}}{\omega_{\mathrm{CG}}^{\mathrm{nor}}C_{\mathrm{CG}}^{\mathrm{nor}}+\omega_{\mathrm{PGB}}^{\mathrm{nor}}C_{\mathrm{PGB}}^{\mathrm{nor}}+\omega_{G4}^{\mathrm{nor}}C_{G4}^{\mathrm{nor}}+\omega_{G5}^{\mathrm{nor}}C_{G5}^{\mathrm{nor}}+\omega_{G6}^{\mathrm{nor}}C_{G6}^{\mathrm{nor}}}\right)\\ \;\frac{d}{\mathrm{dt}}C_{\mathrm{GM}-\mathrm{CSF}}=P_{\mathrm{GM}-\mathrm{CSF}}^{\mathrm{endo}}-\frac{C_{\mathrm{GM}-\mathrm{CSF}}}{T_{\mathrm{GM}-\mathrm{CSF}}} \end{array}$$

#### Granulocyte Colony Stimulating Factor G-CSF

The model includes two G-CSF compartments: *C*_*G-CSF*_ representing blood concentration and *C*_*SC*_ for subcutaneous G-CSF administration which is subdivided into *C*_*SC_1*_ and *C*_*SC_2*_ to model delayed absorption*.*

$$\begin{array}{l} P_{G-\mathrm{CSF}}^{\mathrm{endo}}=Z_{G-\mathrm{CSF}}\left(\frac{{\tilde{\omega }}_{G6}C_{G6}+\omega_{\mathrm{GRA}}C_{\mathrm{GRA}}}{{\tilde{\omega }}_{G6}^{\mathrm{nor}}C_{G6}^{\mathrm{nor}}+\omega_{\mathrm{GRA}}^{\mathrm{nor}}C_{\mathrm{GRA}}^{\mathrm{nor}}}\right)\\ \frac{d}{\mathrm{dt}}C_{G-\mathrm{CSF}}=P_{G-\mathrm{CSF}}^{\mathrm{endo}}+P_{G-\mathrm{CSF}}^{\mathrm{exo}}-\frac{C_{G-\mathrm{CSF}}}{T_{G-\mathrm{CSF}}}\\ \frac{d}{\mathrm{dt}}C_{\mathrm{SC}\_1}=C_{\mathrm{SC}}^{\mathrm{exo}}-k_{\mathrm{SC}}C_{\mathrm{SC}\_1}\\ \frac{d}{\mathrm{dt}}C_{\mathrm{SC}\_2}=k_{\mathrm{SC}}\left(C_{\mathrm{SC}\_1}-C_{\mathrm{SC}\_2}\right)\\ P_{G-\mathrm{CSF}}^{\mathrm{exo}}=k_{\mathrm{SC}}C_{\mathrm{SC}\_2}\\ C_{\mathrm{SC}}^{\mathrm{exo}}=\left\{\begin{array}{l} d_{G-\mathrm{CSF}}\;\\ 0 \end{array}\right)\;\begin{array}{l} t_{i}<t<t_{i}+t_{\inf}\\ \mathrm{otherwise} \end{array} \end{array}$$

Where *t*_*i*_ are the times when the infusions start and *t*_inf_ is the duration of G-CSF application. Degradation is modeled by two independent processes, one dependent on GRA and another independent of GRA [17]:

$$\frac{1}{T_{G-\mathrm{CSF}}}=\frac{1}{T_{G-\mathrm{CSF}}^{\mathrm{ren}}}+\frac{C_{\mathrm{GRA}}^{\mathrm{rel}}}{T_{G-\mathrm{CSF}}^{\mathrm{GRA}}}$$

### A1.2 Model parameter

Here we provide the full set of parameters established by Scholz et.al. [17] and used in the simulations of the ODE model. The identical set was used without any adaptation in the simulations of the hybrid model.

### A1.3 Equilibrium values of all compartments

Evaluation of the parameters given in Table 2 leads to the equilibrium numbers given in Table 3. Please note that these numbers are given in units of equilibrium stem cell number.

**Table 2:** Parameter of the ODE model

**Compartment**	**Parameter**		**value**
S	average duration of cell cycle	*τ*_*S*_	8 h
	amplitude of regulation for probability of self-renewal	*P*_*δ*_	0.1
	minimal proliferative fraction	$a_{S}^{\min}$	0.01
	proliferative fraction at equilibrium	$a_{S}^{\mathrm{nor}}$	0.15
	proliferative fraction for intensified stimulation	$a_{S}^{\mathrm{int}}$	0.45
	maximal proliferative fraction	$a_{S}^{\max}$	1.0
	weight of stem cells in p	ϑ_S_	2
	weight of BM cells in p	ϑ_G_	−10
	weight of stem cells in *a*	ω_S_	1
	weight of bone marrow cells in a	w_G_	0.4
CG	minimal proliferative fraction	$a_{S}^{\min}$	0.3
	proliferative fraction at equilibrium	$a_{S}^{\mathrm{nor}}$	0.33
	proliferative fraction for intensified stimulation	$a_{S}^{\mathrm{int}}$	0.6
	maximal proliferative fraction	$a_{S}^{\max}$	1
	Minimal amplification	*A*^*min*^	1
	equilibrium amplification	*A*^*nor*^	64
	maximal amplification	*A*^*max*^	333
	sensitivity amplification	*b*_*A*_	0.4
	transit time	*T*	112 h
PGB	proliferative fraction	*a*	1
	minimal amplification	*A*^*min*^	4
	equilibrium amplification	*A*^*nor*^	32
	maximal amplification	*A*^*max*^	330
	sensitivity amplification	*b*_*A*_	0.27
	transit time	*T*	148 h
MGB-G4	number of subcompartments	*N*_*G4*_	5
	amplification	*A*	1
	maximal transit time (at minimal cell number)	*T*^*min*^	60 h
	equilibrium transit time	*T*^*nor*^	51 h
	minmal transit time	*T*^*max*^	1 h
	sensitivity transit time	*b*_T_	0.845
MGB-G5	number of subcompartments	*N*_*G5*_	5
	amplification	*A*	1
	transit time	*T*^*min*^	100 h
	transit time	*T*^*nor*^	92 h
	transit time	*T*^*max*^	46 h
	sensitivity transit time	*b*_*T*_	0.845
MGB-G6	number of subcompartments	*N*_*G6*_	5
	minimal amplification	*A*^*min*^	0.01
	equilibrium amplification	*A*^*nor*^	0.4277
	maximal amplification	*A*^*max*^	1
	sensitivity amplification	*b*_*A*_	1.52
	transit time	*T*^*min*^	140 h
	transit time	*T*^*nor*^	22 h
	transit time	*T*^*max*^	20 h
	sensitivity transit time	*b*_*T*_	0.845
GRA	life-time	*T*^*nor*^	5 h
	life time prolongation by Prednison	*T*^*Pred*^	0.66
GM-CSF	maximal production rate	*P*^*max*^	0.91 h^-1^
	equilibrium production rate	*P*^*nor*^	1.0 h^-1^
	minimal production rate	*P*^*min*^	310 h^-1^
	sensitivity production rate	*b*_*P*_	1.7
	life time	*T*^*GM-CSF*^	2 h
	weight of CG	ω_*CG*_	1
	weight of PGB	ω_*PGB*_	1
	weight of G4	ω_*G4*_	1
	weight of G5	ω_*G5*_	1
	weight of G6	ω_*G6*_	0.2
G-CSF	maximal production rate	*P*^*max*^	0.97 h^-1^
	equilibrium production rate	*P*^*nor*^	1.0 h^-1^
	minimal production rate	*P*^*min*^	410 h^-1^
	sensitivity production rate	*b*_*P*_	0.33
	transition rate subcutaneous compartments	*k*_*sc*_	0.75
	administered dose-equivalent	*d*_*G-CSF*_	5.6 · 10^6^
	duration of G-CSF infusion	*t*_*inf*_	2 min
	transit time of unspecific degradation	$T_{{}_{G‒\mathrm{CSF}}}^{{}^{\mathrm{ren}}}$	20 h
	transit time of GRA-specific degradation	$T_{{}_{G‒\mathrm{CSF}}}^{\mathrm{GRA}}$	2.8 h
	weight of G6	ω_*G6*_	0.2
	weight of GRA	ω_*GRA*_	1

**Table 3:** Equilibrium values of all compartments

**CG**^***nor***^	**PRG**^***nor***^	**MGB**^***nor***^	**GRA**^***nor***^	**G**^***nor***^	**GM-CSF**^***nor***^	**G-CSF**^***nor***^
50.4564	828.9625	4633.02	62.1161	5512.4	2.0	2.4383

### A2. Details of the difference model

#### A2.1 Model equations

### Transition functions

The general class of sigmoidal functions used for calculation of transition characteristics and transition intensities is given by:

$$f\left(N\right)=\frac{1}{v_{1}+v_{2}\exp\left(v_{3}\cdot \frac{N}{Ñ}\right)}+v_{4}$$

It is defined by a scaling parameter *Ñ* and four shape parameter *ν*_i_, i = 1,…,4, which can be expressed more intuitively by *f* (0), *f* (*Ñ*_Α_ /2), *f* (*Ñ*_Α_) and *f* (∞) by

$$\begin{array}{l} n_{1}=\left(h_{1}h_{3}-h_{2}{}^{2}\right)/\left(h_{1}+h_{3}-2h_{2}\right)\\ n_{2}=h_{1}-n_{1,}\\ n_{3}=\ln(\;\left(h_{3}-n_{1}\right)/n_{2})\\ n_{4}=f\left(\infty \right), \end{array}$$

with the auxiliary quantities

$$\begin{array}{l} h_{1}=1/\left[f\left(0\right)–f\left(\infty \right)\right]\\ h_{2}=1/[f\left(Ñ/2\right)–f\left(\infty \right)]\\ h_{3}=1/[f\left(Ñ\right)–f\left(\infty \right)] \end{array}$$

#### A2.2 Model parameter

The set of parameters used in the simulations of the hybrid model is given in Table 4. Please note again that this is exactly the same parameter set as used in the leukemia simulations of the ABM except for the small changes in differentiation coefficient and regeneration coefficient [32].

**Table 4:** Parameter of the stem cell compartment in the hybrid model

		
Time step	Δ*t*	1 h
Differentiation coefficient	*d*	1.10252
Regeneration coefficient	*r*	1.05001
Threshold to differentiated cells	*a*_min_	0.002
Maximum stem cell affinity	*a*_max_	1.0
Cycle time	τ_c_	49 h
S/G_2_/M-phase	τ_S_	17 h
Transition Characteristics		
Ω → Α	*f*_Α_(0)	0.5
	*f*_Α_( *Ñ*_Α_ /2)	0.45
	*f*_Α_( *Ñ*_Α_ )	0.05
	*f*_Α_(∞)	0.0
	*Ñ*_Α_	10^5^
Α → Ω	*f*_Ω_(0)	0.5
	*f*_Ω_( *Ñ*_Ω_ /2)	0.3
	*f*_Ω_( *Ñ*_Ω_)	0.1
	*f*_Ω_(∞)	0.0
	*Ñ*_Ω_	10^5^

### A3. Chemotherapy and toxicity parameter

#### A3.1 Schedule of the considered regimen

For quantitative simulations we have considered four established chemotherapy regimen that serve as benchmarks, because they comprise dose- and time-intensification of therapy. Their schedules and cycle as applied in the NHL-B trial [40,41] numbers are provided in Table 5.

**Table 5:** Details of the four considered CHOP-like chemotherapy regimens

**Regimen**	**G-CSF**	**C**	**D**	**V**	**E**	**Pred**	**cycles**
CHOP-21	--	750	50	2	--		6
(398 patients)	--	d1	d1	d1		d 1-5	21d
CHOP-14		750	50	2	--		6
(393)	d4-13	d1	d1	d1		d 1-5	14d
CHOEP-21	--	750	50	2	100		6
(387)	--	d1	d1	d1	d1-3	d 1-5	21d
CHOEP-14		750	50	2	100		6
(398)	d4-13	d1	d1	d1	d1-3	d 1-5	14d

In the table the following abbreviations and related units are used: C: Cyclophosphamide [mg/m2], D: Doxorubicin [mg/m2], V: Vincristine [mg], E: Etoposide [mg/m2], Pred: Prednisone [mg]).

#### A3.2 Toxicity parameter

Scholz et.al. established the toxicity parameters given in Table 6 for their simulations of the four CHOP-like chemotherapy which are summarized in Table 5[17]. We use exactly the same parameters in our hybrid model simulations, including the first cycle effect (*f*_*fc*_ = 1.3).

**Table 6:** Toxicity parameter for simulations of the CHOP-like regimens

**Drug or drug combination**	***k***_***S***_	***k***_***PGB***_	***k***_***MGB***_
C750 + D50 + V2	0.1951	0.5	0.0
E100	0.005	1	0.005

### A4 Preliminary simulation of high-dose chemotherapy with bone marrow transplant

The damage induced by the CHOP-like chemotherapy regimen appears to be small enough to render the feedback from bone marrow cells to stem cells not only unnecessary, but even distorting. For the stronger damage conferred by myeloablative therapies the response of the hybrid model can be accelerated by an adaptation of the transition characteristics that makes them more sensitive to changing cell numbers (Figure 9). This adaptation does not influence equilibrium cell numbers, because values at equilibrium are kept constant.

## Competing interests

The authors declare that they have no competing interests.

## Authors’ contributions

Model development: AK, IR, ML, MS, Design of simulation scenarios: AK, ML, MS, Model simulations and data analysis AK, Paper writing: AK, MS, Contribution to paper writing: IR, ML. All authors read and approved the final version of the manuscript.

## Supplementary Material

Additional file 1 **Pooled patient data of the NHL-B trial of the German High Grade Non-Hodgkin-Lymphoma Study Group for the four considered chemotherapy regimen CHOP-21, CHOP-14, CHOEP-21 and CHOEP-14.** For each of the four regimens, median and quartiles are supplied for each day for the duration of the protocol as given in Appendix, Table 5. Click here for file
